# Tea-residue-derived carbon nanomaterials for adsorption–degradation coupling: mechanistic insights into pollutant removal and sustainability challenges

**DOI:** 10.1039/d6ra01706a

**Published:** 2026-05-11

**Authors:** Shareen Niyazi, Mohammad Shahid, Aman Raj

**Affiliations:** a Department of Environmental Engineering, College of Ecology and the Environment, Nanjing Forestry University Nanjing 210037 China shareen_niyazi@njfu.edu.cn; b Marwadi University Research Center, Department of Agriculture, Faculty of Science, Marwadi University Rajkot 360003 Gujarat India gd4858@myamu.ac.in; c The Zuckerberg Institute for Water Research, The Jacob Blaustein Institutes for Desert Research, Ben-Gurion University of the Negev Sede Boqer Israel

## Abstract

Low-cost adsorption materials derived from waste tea powder (WTPs) have been extensively studied for contaminant removal. However, these materials face limitations such as adsorption site saturation, secondary waste generation, and lack of contaminant removal. Most work in the literature has concentrated on the adsorption efficiency rather than on the catalytic transformation mechanism. Little work has focused on potential applications of WTPs as environmental remediation materials. This review addresses these gaps by examining the transformation of WTPs into multifunctional carbon nanomaterials (CNM) that integrate adsorption and catalytic degradation mechanisms to overcome these limitations. The review highlights the enhanced properties of heteroatom-doped (N, P, S) and defect engineered WTP-derived CNMs, which enable effective activation of peroxymonosulfate (PMS) and peroxydisulfate (PDS) oxidants, facilitating both radical and non-radical pathways for contaminant degradation. In addition, the development of hierarchical porosity and surface functionalization facilitates a capture-degrade mechanism, circumventing the adsorption bottleneck associated with traditional materials. The review describes how WTP-CNMs can be reused in soil systems to help immobilize pollutants, stimulate microbial activity, and advance sustainable remediation, thereby promoting a circular waste-to-resource approach. By linking traditional adsorption-based remediation materials with catalytic-active NMs derived from waste, this review provides design approaches for large-scale and sustainable environmental remediation. Overall, integration of catalytic function with tea-residue-derived NMs represents a viable path for large-scale sustainable environmental remediation.

## Abstract 2

Tea-waste-derived carbon nanomaterials enable sustainable adsorption and catalytic degradation of dyes and emerging pollutants in wastewater treatment systems.

## Graphical abstract

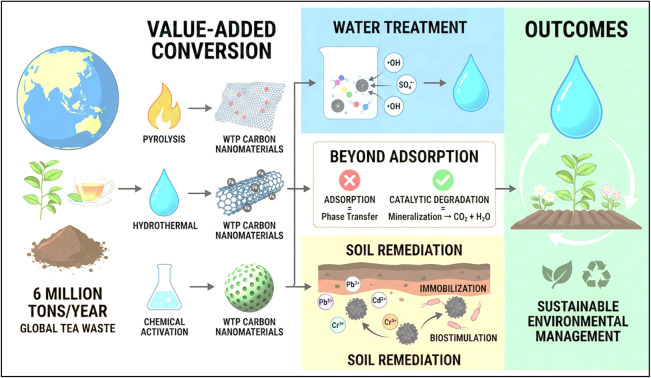

## 1. Introduction

As the tea industry becomes a global agro-business producing more than 70 million tonnes of *Camellia sinensis* each year, there are significant environmental impacts as a result of large amounts of production, with 90% of the processed tea leaves being disposed of as waste.^1,2^ While biodegradable waste is increasingly recognized as a resource for sustainable economic development, effective management strategies at waste treatment plants remain poorly developed. Residual biomass in regional landfills represents hundreds of millions of tonnes, and its decomposition produces greenhouse gases and concentrated organic acids.^3^ On the other hand, non-biodegradable waste streams generated through the biomass disposal require advanced chemical processing. WTPs therefore has significant potential as a feedstock for the manufacture of value-added nanomaterials (NMs) *via* alternative, efficient green chemistry approaches.^4^ The potential of WTPs as a precursor for NMs is determined by its functional groups, such as hydroxyl (–OH), carboxyl (–COOH), and amino (–NH_2_), which facilitate the adsorption of organic and inorganic contaminants during wastewater treatment processes.^5^ WTP generally contains >30% polyphenols (including catechins, flavonoids, and proanthocyanidins) and is rich in lignocellulosic biomass (cellulose, hemicellulose, lignin). Additionally, Liu *et al.* (2023) have shown that WTPs contains 6–8% caffeine and significant amounts of nitrogen (N), phosphorus (P), and potassium (K), and other essential minerals.^6^ However, these components are often produced through regulated agricultural systems and, when disposed of in landfills, may contribute to groundwater pollution or disrupt soil microbial communities.

Additionally, there are many industrial sectors emitting pollutants such as synthetic dye from textiles, leather, and pharmaceuticals manufacturing. Synthetic dyes are a red flag to both marine and terrestrial ecosystems.^7^ Due to their chemical resistance to both fading and degradation by aerobic microbes, synthetic dyes are useful in industrial applications.^8^ Azo dyes, among the most prevalent synthetic dyes, are classified as potential carcinogens and mutagens due to their high levels of toxicity to humans, plants, and aquatic organisms. If untreated, these pollutants enter aquatic ecosystems, increasing the molar excitation coefficients of water and reducing the penetration of visible light.^9^ This reduction in light intensity impairs phytobenthos communities and benthic photosynthetic activity, leading to hypoxic conditions. Such disruptions affect the natural food chain in aqueous ecosystems^10^ and, through the extensive use of dye-contaminated wastewater for agricultural irrigation—specifically in emerging economies—also impact terrestrial ecosystems. As a result, these industrial processes introduce persistent organic pollutants (POPs) into soil systems, resulting in carcinogenic and mutagenic impacts on agricultural ecosystems.^11^ Other drawbacks related to traditional physical and chemical treatment methods include the high cost, lengthy processing times, and the production of secondary sludge.^12^ These challenges underscore the increasing demand for an integrated remediation strategies, such as the utilization of waste biomass like WTPs, to develop eco-friendly and efficient catalysts capable of detoxifying hazardous dyes.^13^

Recent review studies have extensively discussed adsorption-based removal of pollutants and the development of catalytic degradation systems, as well as their combined applications in wastewater treatment.^14,15^ These studies highlight the effectiveness of adsorption and advanced oxidation processes; however, they primarily focus on material performance and process optimization rather than the transformation of biomass-derived waste into multifunctional catalytic NMs. The recent focus of research on water treatment has begun to explore the potential of combining adsorption with advanced oxidation processes (AOPs), so that pollutants can be adsorbed and also degraded using radicals produced through these two processes.^16,17^ This represents a new and innovative way of addressing the issues of conventional treatment technologies. In *RSC Advances*, recent studies have detailed the use of carbon nanomaterials (CNMs) and nanocomposites to achieve efficient adsorption and catalytic degradation of the pollutants, with an emphasis on the effects of surface functionalization, heteroatom doping and defect engineering to improve performance.^18–21^ While other types of biomass-derived NMs may be occasionally cited for comparison purposes, the primary focus of this review will be on the use of tea-residue-derived CNMs to maintain a thematic focus and increase the relevance of this review to the subject matter presented.

In order to ensure clarity and consistency, this review specifically interpret CNMs derived from tea-residues-derived NMs. Though references to other biomass-derived materials will also be incorporated into this review for comparison, the selection of studies included in this review will focus mainly on investigations that are directly related to WTPs systems. Thus, non-WTPs studies will be included only as a means of supporting methodology and interpretation of mechanisms. Unlike many research publications, this review extends beyond traditional adsorption-based studies of tea waste materials by emphasising their transformation into multi-functional CNMs with catalytic properties. Therefore, there are four primary objectives pertaining to this review, which are as follows: (i) to systematically evaluate synthetic methods for generating CNMs from WTPs; (ii) to critically compare the mechanisms of adsorption and catalytic degradation; (iii) to delineate how material desing features such as heteroatom doping and porosity enhance performance; and (iv) to investigate the use of CNMs derived from WTPs for aqueous and soil remediation. Furthermore, this review addresses current literature gaps, including the limited understanding of the adsorption–degradation coupling, insufficient mechanistic insight into catalytic reaction pathways, and lack of integrated approaches linking water treatment and soil remediation.

This structure of this review is as follows: Section 2 discusses various methods for synthesizing CNMs from WTPs. Section 3 examines adsorption mechanisms and their limitations. Section 4 addresses catalytic degradation pathways of CNMs and material design. Section 5 outlines applications of CNMs in soil remediation, and Section 6 proposes future research directions. This report offers a comprehensive overview of CNMs derived from tea residue, highlighting their interrelation with adsorption limitations, catalytic degradation pathways, and soil remediation applications within a unified framework.

## 2. Review methodology

A structured literature search was employed to ensure comprehensive and relevant coverage of the topic. Scientific databases, including Web of Science, Scopus, and Google Scholar, were used to identify relevant publications. The search was performed using combinations of keywords such as ‘tea waste’, ‘tea residue’, ‘carbon nanomaterials’, ‘biochar’, ‘adsorption’, ‘catalytic degradation’, and ‘advanced oxidation processes. The literature search primarily targeted studies published between 2010 and 2025, with particular emphasis on recent advancements. Inclusion criteria prioritized studies reporting the synthesis, characterization, and environmental applications of tea-residue-derived carbon materials. Studies focusing solely on non-tea biomass-derived materials were included only for comparative or mechanistic insights. Exclusion criteria involved studies lacking experimental validation, insufficient characterization, or unrelated applications. To maintain the scope of the review, special attention was given to distinguishing WTP-derived CNMs from general biomass-derived carbons, thereby ensuring a focused and thematically consistent discussion. The selected studies were critically analyzed to identify trends, limitations, and research gaps in adsorption–degradation systems.

## 3. Waste-tea-derived powder (WTP) carbon nanomaterials (CNMs)

### 3.1 Why WTPs is an ideal sustainable carbon precursor

High levels of bioactive compounds and non-native heteroatoms, such as N, in lignocellulosic biomass from WTP provide a robust foundation for the synthesis of advanced CNMs.^1^ Unlike most traditional agricultural residues, which often require either harsh chemical activation or the addition of foreign dopants, WTP serves as a single-source precursor for CNMs development. Tea polyphenols, including catechins, theaflavins, and phenolic acids constitute 30–40% of the dry weight of WTPs biomass.^22^ These polyphenols possess abundant phenolic hydroxyl groups, which function as efficient reducing and capping agents during reactions with metal ions, resulting in the formation of stable NMs.^23^ Recent studies demonstrate that the agglomeration of NMs synthesised from WTP-based waste biomass can be effectively controlled by utilizing the complex aromatic structure of the final product, thereby maintaining a high surface area-to-volume ratio necessary for efficient degradation.^24^ As a result of its inherent self-doping capability, WTPs is considered as one of the most effective carbon precursors available.^25^ Compared with other biomass sources, WTP exhibits a high N content, attributable to the presence of alkaloids such as caffeine, amino acids like l-theanine, and structural proteins.^26^ During hydrothermal carbonization or pyrolysis, these nitrogenous compounds are incorporated into the carbon structure as pyridinic, pyrrolic, and graphitic N.^27^ N-doping enhances pollutant detoxification by introducing positive charge centres and generating active defect sites within the carbon matrix. These modifications improve the electronic conductivity of carbon and facilitate the activation of oxidants such as PMS and PDS.

WTP-generated NMs are characterized by a stable matrix containing various types of pores formed from diverse lignocellulosic material.^4^ The pyrolysis process yields a robust carbon skeleton derived from lignin present in WTP. Decomposition of hemicelluloses during pyrolysis results in the formation of both micropores and mesopores within the NMs.^28^ The micropores provide a large surface area for interaction between dye molecules and the NMs, while the mesopores facilitate efficient transport to catalytic sites.^29^ The distinct chemical compositions of WTP materials enable the development of advanced and efficient catalysts from industrial waste, thereby promoting the principles of green chemistry and the circular economy.^30^

### 3.2 The limitation of traditional WTP adsorbents

The conventional application of WTPs for environmental remediation *via* adsorption does not offer a comprehensive solution; rather, it primarily relocates pollutants from one location to another.^31^ In contrast, raw tea waste and activated carbon (AC) derivatives produced form tea waste demonstrate high efficacy in sequestering a wide variety of synthetic dyes and heavy metals, including Pb, achieving estimated removal efficiency of >95%.^32^ The preservation of toxic molecular integrity during physical or chemical binding underpins the mechanism for contaminant sequestration. This section outlines the technological and ecological bottlenecks that extend beyond simple adsorption. One major bottleneck is the generation of spent adsorbents, which are solid waste materials saturated with highly concentrated pollutants.^33^ At large-scale industrial sites, such as tea factories where up to 30 000 tons of spent adsorbent is produced annually, the safe disposal of toxic pollutants presents a substantial barrier to the practical use of these materials.^34^ Unlike raw tea waste, which is biodegradable, a spent adsorbent pose potential threats to human health and safety. Improper disposal, such as landfill dumping or open dumping into rivers and other water bodies, is likely to result in secondary pollution through the leaching of previously sequestered chemicals.^35^

The technical limitations of conventional water treatment plant adsorbents have restricted their long-term viability.^36^ Brunauer–Emmett–Teller (BET) analysis has demonstrated significant reductions in surface area, primarily due to irreversible pore blockage by large dye molecules.^37^ To enhance the quality of AC derived from tea waste-based products, chemical activation agents such as sulfuric acid, zinc chloride, or orthophosphoric acid may be employed.^31^ These agents promote the formation of porous structures and increase the chemical properties of the production process. During biomass thermal degradation, exposure to high temperatures (>550 °C) results in complete conversion of biomass into ash, while lower temperatures lead to partial carbonization, thereby reducing both the effectiveness and yield of adsorbents.^38^ Traditional adsorption methods does not meet its intended goal of zero-waste remediation cycles due to its failure to mineralise complex organic dyes into environmentally benign CO_2_ and H_2_O.^39^ To advance zero-waste strategies, current research should focus on developing multi-functional materials incorporating rapid adsorption capabilities with WTP systems and efficient catalytic degradation.

### 3.3 Scope, novelty, and objective of this review

**Fig. 1:**
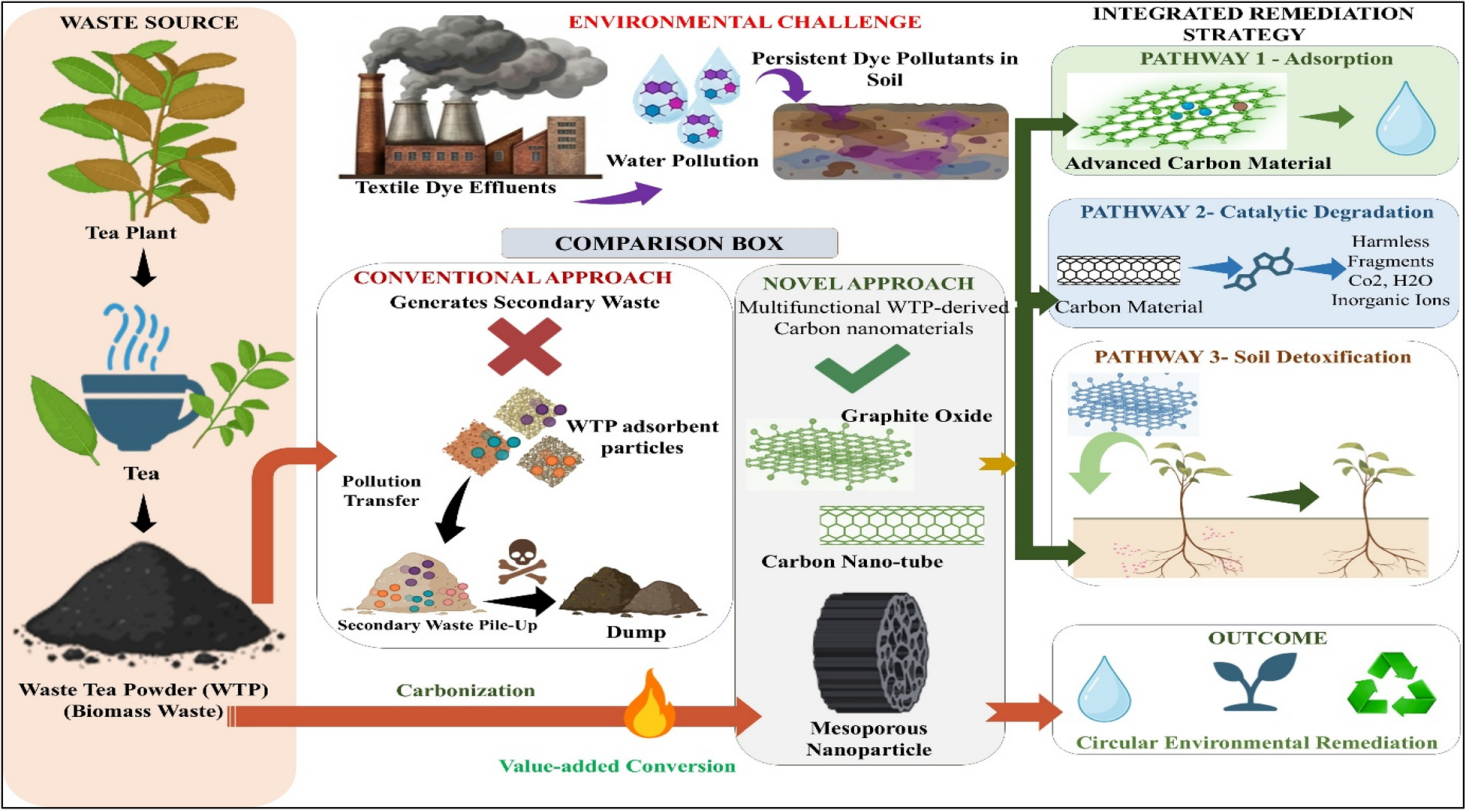
Conceptual framework illustrating the beyond adsorption paradigm of WTP-derived CNMs. Global tea consumption generates large quantities of waste tea powder (WTP), while persistent dye pollutants from textile and allied industries contaminate water and soil systems. Conventional WTP-based adsorbents merely transfer pollutants from water to spent solids, creating secondary waste streams. In contrast, multifunctional WTP-derived CNMs enable an integrated remediation strategy by combining adsorption, catalytic degradation, and soil detoxification, thereby transforming waste biomass into a value-added material for circular environmental remediation.

This review examines the role of WTPs as a passive bio-adsorbent, highlighting its potential as a precursor for multi-functional CNMs. The discussion focuses on materials which serve three primary functions: rapid adsorption, catalytic mineralization, and potential soil detoxification. Previous research has primarily addressed pollutant removal using unaltered tea debris, which has attracted considerable attention. The novelty of this review originates from its integrated perspective, positioning WTP-derived CNMs not only as a water treatment solution but also as a hybrid remediation tool that bridges the gap between aquatic ecosystem restoration and soil recovery. As shown in Fig. 1, this approach moves beyond the traditional linear ‘use-and-discard’ model for tea waste-derived adsorbents, proposing a circular ‘capture-kill-restore’ system that integrates water remediation with soil health improvement.

This review addresses four key aspects of environmental technology:

(1) To comprehensively determine the role of WTP-CNMs in transitioning from passive sequestration agents to active catalytic structures, with particular emphasis on the effects of intrinsic heteroatom doping, including N, P, and K.

(2) To thoroughly investigate the electronic and structural mechanisms, including both radical and non-radical pathways, that enable WTP-CNMs to activate PMS and PDS, which catalyze heterogeneous Fenton reactions.

(3) To evaluate the adsorption bottleneck and validate the capture-and-kill model as an alternative to the traditional linear adsorption–disposal cycle.

(4) To assess post-remediation management strategies for WTP catalysts, exploring their potential role as soil conditioners or biostimulants to enhance microbial activity in polluted terrestrial ecosystems.

## 4. From waste to nano: fabrication and structural control

The formation of CNMs using WTPs during biomass valorisation represents an advanced strategy for developing value-added functional materials, offering advantages over traditional waste management approaches.^2^ The synthesis of CNMs is attributed to the complex biochemical composition of the tea feedstock, including lignocellulosic biomass and other bioactive compounds such as polyphenols and alkaloids. WTPs serves as a source of carbon and natural heteroatoms such as N, S, and P, as well as oxygen-rich functional groups. These components function as *in situ* dopants and stabilizing agents, enabling the synthesis of self-doped nanostructures without the need for toxic precursors.

### 4.1 Synthesis routes for WTP-CNMs

The fabrication of CNMs from WTPs represents a novel approach in molecular tailoring. The synthesis protocol critically influences whether the resulting NM functions as a passive adsorbent or as an active catalyst. Table 1 summarizes the various synthetic routes, physicochemical properties, and the complex surface chemistry derived from WTPs. The current literature distinguishes between “top-down” thermal pathways and “bottom-up” chemical approaches, each utilizing distinct bioactive compounds in the tea matrix.^49^ Among these, hydrothermal carbonization (HTC) is the most widely reported ‘bottom-up’ approach for producing carbon dots (CDs) and carbonaceous microspheres.^50^ Previous studies have demonstrated that HTC decomposes hemicellulose and soluble polyphenols in tea residues at temperatures ranging from 250 °C and 400 °C in an aqueous environment.^51^ Self-doping is a key feature of HTC. During the nucleation phase, the natural N and S content in tea proteins and alkaloids becomes embedded into the carbon lattice.^52^ This process yields highly fluorescent, N, S-co-doped CDs with a high density of surface functional groups. These features, often lost in higher-temperature processes, are essential for applications in sensing and catalytic degradation of recalcitrant dyes such as gefitinib or methylene blues (MB).^51,53^

**Table 1:** Synthesis routes, physicochemical properties, and surface chemistry of WTP–derived CNMs

Synthesis route (precursor → treatment)	Activation/agent	Temp./conditions (as reported)	Reported surface area (m^2^ g^−1^)	Pore structure (authors' description)	Dominant surface functional groups (FTIR/XPS summary)	Natural doping/metals reported	References
Hydrothermal carbonization of tea waste → KOH activation (samples TAC, TAC2/3/4)	KOH (different precursor: KOH ratios)	HTC 180 °C (10 h) → KOH activation 800 °C (N_2_)	TAC3: 2235 m^2^ g^−1^	Hierarchical micro/mesoporous; interconnected pores (HTC → KOH creates hierarchical network)	Abundant O-containing groups (–OH, –CO, C–O); some C–N features reported	N, O (heteroatom doping from precursor)	40
Pre-carbonization + KOH activation of tea woody fraction	KOH	Activation (700–800 °C)	∼1610–2559 m^2^ g^−1^ (different samples; 2559 m^2^ g^−1^ for highly porous AC; micropore area ∼1614 m^2^ g^−1^)	Highly microporous dominated by micropore area; hierarchical pores in optimized sample	Graphitic domains + oxygenated surface groups after activation	Ash/inorganics reported (woody part contains minerals)	41
Ultrasound-assisted two-step NaOH impregnation + carbonization (tea leaves → TEA (char)-AC)	NaOH (sonication + impregnation)	Carbonization/activation at 650 °C (N_2_)	1151 m^2^ g^−1^ (TEA (char)-AC)	Combined microporous + small mesopores; high micropore fraction	O-containing groups (–COOH, –OH); some basic N-species depending on precursor	N and O from tea precursor; ash content reported	42
H_3_PO_4_ chemical activation of tea waste (various atmospheres)	H_3_PO_4_	Activation/carbonization (varied; often 500–800 °C)	∼785–880 m^2^ g^−1^ (H_3_PO_4_-AC reported 880 m^2^ g^−1^)	Micropore + mesopore character; H_3_PO_4_ promotes mesopore formation and surface acidity	Phosphate-bonded surface moieties, C–O, C=O; acidic oxygen functionalities	N, O; inorganic ash depending on feedstock	43
Physical activation (steam/CO_2_) of pre-carbonized tea waste (steam activation)	Steam/CO_2_ (physical)	Carbonization then activation at 700–900 °C (oxidizing gas)	∼<100–400 m^2^ g^−1^ typically reported for physical activation of tea waste	More mesopore development with steam/CO_2_; lower SSA than aggressive chemical activation	Condensed aromatic C; fewer oxygenated surface groups *vs.* chemical activation	Residual ash/metal traces from feedstock	44
Simple pyrolysis (slow pyrolysis) of tea waste → biochar (no chemical activation)	None	Pyrolysis 300–700 °C (varied studies)	Very low SSA: *e.g.*, 5–15 m^2^ g^−1^ (authors report single-digit to few-tens m^2^ g^−1^ for non-activated biochars)	Poorly developed porosity; mainly condensed aromatic carbon; small micropore population	Some O-containing surface groups (dependent on T); condensed aromatic signals at higher T	Inherent N and mineral traces partly retained	45
Metal-modified tea biochar/AC (*e.g.*, Fe/Mn/La impregnation)—pyrolysis then metal addition or *in situ* impregnation	Fe, Mn, La salts (post-treatment or *in situ*)	Pyrolysis 500–800 °C; metal impregnation conditions vary	Biochar/AC SSA varies: 140–840 m^2^ g^−1^ (metal-modified samples	Porous biochar with embedded/anchored metal (often increases functional sites; sometimes reduces SSA relative to parent AC)	Metal–oxygen bonds; increased surface –OH, –COOH; metal complexes	Immobilized metals such as Fe, Mn, La, Mg, Cu reported	46
Low-temperature KOH activation of tea twig waste (energy-saving approach)	KOH (mild activation temperatures reported in some newer studies)	Activation at reduced T (reported steps 200–300 °C KOH reactions in some novel protocols)	Varied; representative HT-KOH reports show several hundred → >1000 m^2^ g^−1^ depending on conditions	Micropore/mesopore depending on activation severity; milder treatment → mesopores & lower micropore fraction	Oxygenated groups with preserved hydrochar-like groups	N, O doping from precursor	47
Two-stage process: hydrochar + templating agents (hard/soft templates such as silica or ZnO) → template removal	Templates (SiO_2_, ZnO) ± chemical activation	HTC/carbonization then template removal (acid/base wash); templating temps vary (500–900 °C)	Hundreds → >1500 m^2^ g^−1^ reported for templated porous carbons from tea waste	Highly ordered mesoporous structures possible (with controlled pore sizes from template)	Rich surface functionalities (–COOH, –OH) plus heteroatoms	Possible immobilized metal traces (if metal templates used); N & O doping from precursor	48

**Fig. 2:**
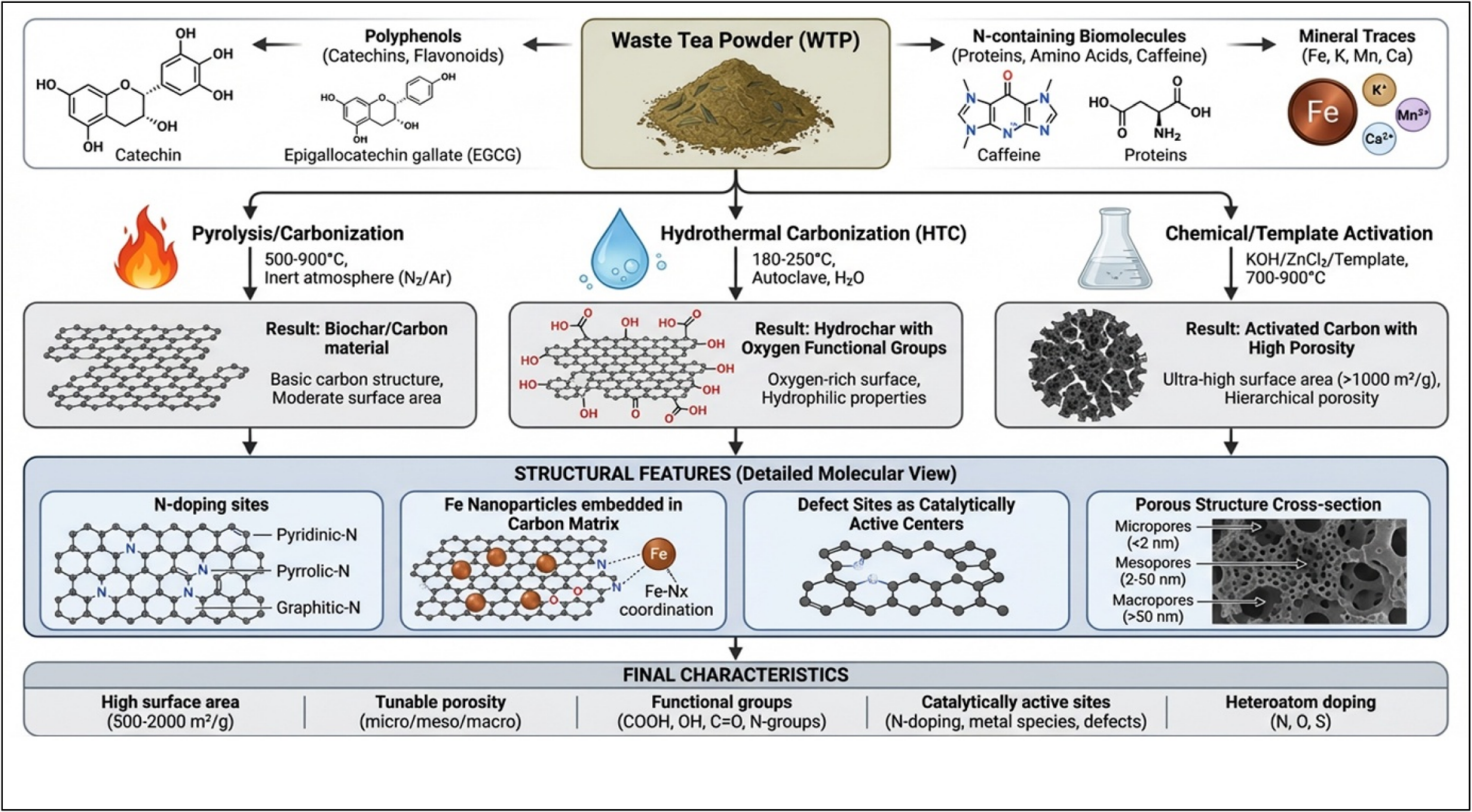
Synthesis routes and structural engineering of WTP-derived CNMs enabling multifunctionality. Different fabrication pathways—pyrolysis/carbonization, hydrothermal carbonization, and chemical or template-assisted activation—convert WTP into structurally diverse CNMs. The intrinsic chemical composition of WTP (polyphenols, nitrogen-containing biomolecules, and mineral traces) facilitates *in situ* heteroatom doping, defect formation, and immobilization of metal species. These features collectively generate high surface area, tunable porosity, abundant functional groups, and catalytically active sites critical for adsorption, oxidation, and soil remediation.

During ‘top-down’ pyrolysis and carbonization process, the structural and electrical conductivity of the carbon lattice are favoured over surface functionality.^54^ The dual transformation pathways of WTPs into porous biochar *via* the top-down approach and fluorescent CDs *via* the bottom-up approach demonstrate the significant influence of temperature on the morphology of the resulting NMs, as shown in Fig. 2. The HTC process produces discrete NMs, whereas pyrolysis typically yields bulk biochar or graphitic structures.^55^ At high temperatures between 500 °C to 900 °C, lignocellulosic biomass undergoes extensive aromatic ring condensation under inert conditions.^56^ Further research is needed to elucidate the “Functionality-Porosity Trade-Off”.^57^ High temperatures enhance surface area and pore volume by thermally decomposing organic matter, thereby enhancing suitability for traditional adsorption applications.^58^ This decomposition also removes heteroatoms, which serve as catalytic sites for mineralization.^59^ Consequently, research is increasingly focusing on two-stage synthesis: an initial HTC phase that incorporates heteroatoms into a stable precursor, followed by mild carbonization to enhance conductivity and porosity. This approach yields hybrid material capable of both rapid adsorption and electron-relay catalysis.

Waste tea extracts serve as ‘bio-factories’ for synthesizing metal–carbon hybrids, representing the most complex synthesis route.^60^ The aqueous extract is rich in the polyphenol epigallocatechin gallate (EGCG), which acts both as a reducing agent for metal salts and a stabilizing capping agent during the formation of silver NMs.^61^ This localized fabrication prevents a secondary contamination commonly associated with traditional chemical reductants such as NaBH_4_. The resulting AgNM-carbon composites act as both adsorbents and green catalysts, utilizing the high surface energy of silver sites to accelerate the dye degradation in the presence of oxidants such as H_2_O_2_.^62^ Comparative analysis indicates that HTC is optimal producing surface-active quantum-scale materials, while pyrolysis is more suitable for fabricating robust porous frameworks. The implementation of environmentally friendly metal reduction represents a significant advancement in multifunctionality, transforming the waste problem into a modern tool for terrestrial and aquatic remediation.

### 4.2 Natural doping and defect engineering

The efficiency of WTPs as a precursor for multi-functional CNMs is rooted in its inherent elemental complexity, which facilitates *in situ* heteroatom doping.^63^ Unlike synthetic carbon precursors, which require the addition of urea or thiourea to introduce catalytic sites, WTP serves as a naturally enriched reservoir of N, P, and S, and various trace minerals.^64^ N, the most critical dopant for environmental catalysis, is naturally present in tea biomass in the form of caffeine, theanine, and structural proteins.^65^ During thermal processing, nitrogenous molecules undergo cyclization and condensation, integrating directly into the developing sp^2^ carbon structure as pyridinic, pyrrolic, and graphitic N sites.^66^ XPS analysis showed a high fluorescence quantum yield of 14.8% and localized charge redistribution.^51^ These redistributions establish a “push–pull” electronic mechanism in which electronegative N atoms withdraw electrons from adjacent carbon atoms.^67^ Consequently, the resulting cationic active centres facilitate the adsorption and catalytic reduction of anionic contaminants.

Mineral trace elements present in WTPs, such as Fe, Al, and Mg, offer distinct advantages for introducing heteroatoms *via* substitution reactions.^68^ The tea plant absorbs trace amounts of these metals from the soil, supplying elements that act as internal catalysts during carbonisation process. Previous studies on biomass valorisation highlight the critical role of these trace minerals in forming localised clusters of graphite-like sheets and M–N–C active sites during carbonisation.^69^ Trace metals, including Fe and Mg, applied in AOPs, enhance the generation of reactive oxygen species (ROS). The carbon matrix prolongs the operational lifespan of metal-loaded catalysts by preventing leaching during self-mineralisation, thus enabling the carbon structure to function as a heterogeneous catalyst. The “Beyond Adsorption” methodology is attainable through defect engineering achieved *via* natural doping. The high ID/IG ratio from Raman spectroscopy indicates that S and N act as synergistic co-dopants, leading to disordered carbon.^51^ Defect sites introduce high-energy edge sites, disrupting the typical hexagonal arrangement of graphene-like sheets. These sites enhance electron transfer from the carbon surface to dye molecules and to oxidants such as PMS and PDS, which serve as electron relay catalysts. Utilizing CNM derived from natural impurities in WTPs enables the removal of dye molecules *via* electrostatic interactions, while simultaneously producing CO_2_ and H_2_O through catalytic dye decomposition. This approach reduces secondary waste typically generated by traditional adsorption methods.

Recent studies investigating topological defects, such as pentagon and heptagon pairings, and molecular dopants, including heteroatoms, have revealed a distinctly different electronic landscape for multifunctional remediation in WTP-derived CNMs. Defect engineering in WTP-CNMs, as opposed to traditional ACs, emphasizes surface quality rather than solely pore quantity. Trace minerals present in carbonized tea residue, such as aluminium (Al) and iron (Fe), function as Lewis acid sites, thereby, reducing the activation energy required for the breakdown of complex dye bonds.^70^ Under varying environmental conditions, including changes in pH, WTP-derived NMs demonstrate superior performance compared to synthetic materials.^71,72^ Consequently, the structural defects in carbon produced from tea residues enhance the decomposition of synthetic pollutants that are resistant to conventional bio-adsorption mechanisms.

### 4.3 Key structural characteristics driving multi-functionality

Structural modifications enable the material to perform adsorption, sensing, and demonstrating the transformation of WTPs from a waste material into high-value-added NMs. These multifunctional properties arise not from carbonisation alone, but from the synergistic effects of hierarchical porosity, extensive functionalised surface area, and a distinct core–shell electronic configuration. According to Hassan and Carr (2021), WTP-derived carbon forms a hierarchical pore structure, with macropores (>50 nm) serving as the primary channels for rapid transport of large dye molecules into the material's interior.^57^ Once inside, mesopores and micropores provide a high-surface-area internal space (greater than 1000 m^2^ g^−1^ when activated) necessary for efficient sequestration. This structure ensures that reaction species can access internal surfaces even after the external surface is saturated, thereby preventing the formation of blocked pores, a common cause of low performance in biomass-derived materials.

The “Beyond Adsorption” theory posits that, in addition to their ability to adsorb a wide range of dye molecules, WTP-CNMs are effective for dye remediation due to their high density of reactive functional surface groups, which actively engage in the remediation process. The presence of these surface functional groups is confirmed by FTIR and XPS, which indicate that WTP-CNMs possess abundant –OH, –COOH, and –NH_2_ groups.^73,74^ These functional groups serve as electrostatic anchors for cationic dye molecules *via* ionic exchange and provide sites for hydrogen bonding and π–π interactions, which are primary mechanisms for the decolorization of persistent dyes such as Congo Red.^75^ WTP-CNMs function as “molecular sponges” with chemical functionalities that can be engineered or tuned to target specific contaminants in complex textile wastewater, unlike inert commercial carbons. This includes the attachment of cationic dyes *via* electrostatic interactions and the formation of bonds through hydrogen bonding and π–π interactions.^75^ Specifically, tea residue-derived CDs exhibit multifunctionality due to their “core/shell” electronic structure.^76^ Studies have demonstrated that CDs derived from tea residues possess a crystalline graphitic carbon core surrounded by an amorphous matrix of heteroatom-doped materials.^77^ The carbon core acts as an electron reservoir, while the defect-rich shell facilitates electron transfer from the graphitic surface to the dye or oxidant molecules.^78^ The high levels of N and S in the shell layer enable the formation of chemical bonds with the electronegative moieties of dye or oxidant molecules, thereby facilitating light detection *via* CD-dye interactions. The unique core/shell electronic structure allows tea-derived CDs to integrate traditional adsorption with active optical or photocatalytic pathways for pollutant removal. Additionally, the structure of tea-derived CDs enables not only adsorption of pollutants but also the generation of ROS for further degradation of contaminants.^79,80^

The robust structural formation of tea-derived material contributes to its stability within a circular economy. Osman *et al.* (2022) report that capability of NMs to maintain more complex structures than rGO enhances the composite material's durability, attributed to their superior thermal and mechanical properties.^81^ This structural toughness enables the material to be recovered and reused multiple times with minimal degradation. In addition, the presence of organized transport channels, a high density of reactive chemical sites, and a catalytic core–shell interface collectively transform WTPs from a simple waste product into a complex, multi-functional tool for detoxification in both the aquatic and terrestrial environments.^82^

## 5. Adsorption performance: mechanisms and limitations

Adsorption is a surface-driven process in which pollutants are captured onto the adsorbent through physical or chemical interactions without altering their chemical structure. While adsorption is effective for rapid pollutant removal, it does not result in pollutant degradation, leading to saturation and secondary waste generation.^83^

### 5.1 Adsorption mechanisms of cationic *vs.* anionic dyes

The difference between cationic and anionic dye adsorption using WTP-derived NMs is primarily governed by electrostatic interactions related to the point of zero charge (pH_PZC_).^84^ Adsorption is particularly efficient for cationic dyes, such as MB and gentian violet, under neutral to alkaline conditions.^85,86^ When the pH increases to 7.05, exceeding the pH_PZC_ of tea waste, surface functional groups, including –COOH and –OH, undergo deprotonation to form –COO– and –O– ions. Consequently, the positively charged cationic dye molecules are strongly attracted to negatively charged adsorbent surface through electrostatic forces. Additionally, π–π interactions occur between the graphitic layers of the adsorbent and the aromatic rings of the cationic dyes, facilitated by the aromatic structure of the carbonized tea matrix.^87^ Hydrogen bonding between the N atoms of the dye and the oxygenated groups of the adsorbent further stabilizes the adsorption process.

A significant limitation of unaltered tea waste is electrostatic repulsion during the adsorption of anionic dyes, including methylene orange (MO), acid blue 25 (AB25), eriochrome black T (EBT) and reactive black 5 (RB5).^88,89^ When the pH exceeds the pH_PZC_, both the adsorbent surface and the anionic dyes acquire negative charges, which restricts removal efficiency due to kinetic constraints. Surface modification and acidification are necessary to facilitate electrostatic binding or anion exchange. Contrarily, at pH values below the pH_PZC_, protonation of surface functional groups (such as –OH_2_^+^ and –NH_3_^+^) occurs.^90^ This generates a positively charged surface that attracts negatively charged sulfonic acid groups (–SO_3_^−^) present in anionic dyes.^91,92^ Previous studies have employed polyethyleneimine (PEI) to convert tea residue into a strong anion-exchange material by inducing basic N-containing surface groups that retain a positive charge across a wide pH range.^93^

The progression of the “Beyond Adsorption” transition is more clearly defined in this context. Effective detoxification of anionic dyes is typically achieved by introducing surface defects or by employing controlled modifications that enhance electronic coupling between the cationic dye and the substrate (WTPs).^94,95^ For both uncarbonized (raw) and carbonized (final) forms of WTPs, the oxygen-rich surfaces facilitate the immobilization of cationic dyes.^96^ Although adsorption remains the primary method for contaminant removal from WTPs, increasing extent or frequency of structural modifications, such as amino doping with NaNH_2_ or the creation of pyridinic N sites, is crucial.^97^ These findings suggest that the material not only serve as an inactive reservoir for cationic pollutants but also functions as a selective active material.^98,99^

### 5.2 Critical comparison of WTP-CNMs to commercial adsorbents

WTP configurations for commercial activated carbon (CAC) commonly employ three primary material types: coal-based, coconut shell, and hardwood sources such as mahogany and red oak. Research on CAC demonstrates that its high surface area is a significant advantage. In comparison, WTP-derived materials typically exhibit an even greater surface area, which enhances their effectiveness in treating the complex chemistry of contemporary industrial wastewater. Table 2 summarizes the differences in adsorption capacities and kinetics for cationic and anionic dye removal using WTP-derived materials.

**Table 2:** Adsorption capacity and kinetics of WTP-derived CNMs for removal of cationic and anionic dyes

Dye type	Dye name	CNM/adsorbent (from tea waste)	Adsorption capacity *q*_max_ (mg g^−1^)	Best-fit isotherm model	Kinetic model (best fit)	pH range studied	Regeneration cycles (retention)	References
Cationic	Methylene blue	Raw tea waste (TW)	85.16	Langmuir	Pseudo-second-order	2–10	Not reported	100
Cationic	Methylene blue	NaOH-modified rejected tea (N-RT)	242.11	Langmuir	Pseudo-second-order	2–10	Not reported	101
Cationic	Methylene blue	Thermally treated green tea waste (TTGTW500)	∼69.0	Freundlich	Pseudo-second-order	2–10	3 cycles (≈80–85% retained)	102
Cationic	Methylene blue	H_3_PO_4_-activated tea-waste AC (H-AC)	∼45.66	Freundlich/Langmuir	Pseudo-second-order	3–10	4 cycles (retention reported)	53
Cationic	Crystal violet	Tea dust (TD)	175.4	Langmuir	Pseudo-second-order	∼pH 7 optimal	Not reported	103
Cationic	Crystal violet	Tea waste/Fe_3_O_4_ magnetic composite (TWMC)	333.33	Langmuir	Pseudo-second-order	pH 7 optimal	Not reported (stability tested)	104
Anionic	Congo red	Raw tea waste (batch tests)	32.26 → 43.48	Langmuir	Pseudo-second-order	2–8	Not reported	105
Anionic	Congo red	CTAB-modified tea waste	Up to ∼200 mg g^−1^	Langmuir/Freundlich	Pseudo-second-order	2–8	Not reported	106
Cationic	Malachite green	Activated carbon from spent tea (STAC)	256.4	Langmuir	Pseudo-second-order	pH ∼4 optimum	3 cycles (high retention)	107
Cationic	Malachite green	Tea stalk powder (unmodified)	∼91.45	Langmuir/Freundlich	Pseudo-second-order	pH 4–7	Not reported	108
Cationic	Rhodamine B	Used black tea leaves (UBTL)	53.2	Langmuir	Pseudo-second-order	pH 2–6	Not reported	109
Anionic	Acid orange/acid blue-type (*e.g.*, acid blue 25)	Tea-derived activated carbon (chemically activated)	∼285	Freundlich/Langmuir	Pseudo-second-order	2–6	3 cycles	110
Mixed/Reactive	Reactive black 5	Tea-waste activated carbon/modified TW	∼198	Langmuir	Pseudo-second-order	3–9	3 cycles	111
Cationic	Safranin-O/similar basic dyes	Tea-derived biochar/activated tea adsorbent	∼148	Langmuir	Pseudo-second-order	5–10	3 cycles	112

Various forms of CAC have been investigated for their efficacy in removing dyes from wastewater.^113,114^ For instance, mahogany sawdust-derived carbon demonstrated adsorption capacities of 518 mg g^−1^ for Direct Blue 2B and 327.9 mg g^−1^ for Direct Green B, both conforming to the Langmuir adsorption model.^115,116^ CMK3 (Carbon Material Kinetic-3) achieved complete removal of methyl orange (MO) within 60 min.^117,118^ Acidic media resulted in a higher percentage of dye removal compared to basic media, and increasing the initial dye concentration enhanced adsorption. Equilibrium data were well described by the Langmuir isotherm, with an adsorption capability of 294.1 mg g^−1^ at 25 °C. AC-derived from apricot stones exhibited adsorption capacities of 36.68 mg g^−1^ for MB and 32.25 mg g^−1^ for MO at pH values of 4.85 and 4.87, respectively, both characterized by the Langmuir isotherm.^119,120^ ACs synthesized from red oak (*Quercus rubra*) effectively removed MB with an efficiency of 97.18%.^121^ The adsorption capacities of three commercial ACs—Norit Darco 12 × 20 [DARCO], Norit R008 [R008], and Norit PK13 [PK13] s—for Reactive Black 5 after 24 hours were 348 mg g^−1^, 527 mg g^−1^, and 394 mg g^−1^, respectively; these results were well described by the Langmuir, Freundlich, and Langmuir–Freundlich models.^122,123^ AC produced from spent tea leaves (STACs) also effectively removed malachite green (MG), with an adsorption capacity of 256.4 mg g^−1^ at 45 °C. Collectively, these studies indicate that the removal of MG from water by adsorption increases with pH up to 4, after which the removal rate remains constant.^107,124^ It should be noted that these results are derived from general biomass-based systems and may not directly reflect the behavior of tea-residue-derived CNMs.

The application of NMs is vital for real-time bio-imaging analysis and precision drug delivery, as highlighted in recent studies.^125,126^ Conventional synthesis methods for these materials are limited by high costs, hazardous reagents and complex sequential pathways.^127^ Consequently, there is a growing demand for biosynthesis processes that avoid toxic precursors and utilize tea waste, which retains the necessary chemical and physical properties for large-scale production.^128^ Tea waste has proven to be an effective, low-cost adsorbent capable of removing contaminants, including toxic dyes, polycyclic aromatic hydrocarbons, antibiotics, and pharmaceutical residues from wastewater.^128,129^ Furthermore, tea waste precursors have been shown to be beneficial for the green synthesis of carbon dots (CDs).^130,131^ CDs exhibit unique photophysical properties, enabling applications in pharmacology, biosensors, medical imaging and environmental monitoring.^132^ Additional advantages of CDs include high water solubility, low toxicity, stable fluorescence, scalable synthesis, compatibility, and numerous surface groups for ligand conjugation.^133^ Most current methods for synthesizing fluorescent CDs rely on traditional carbon sources and chemicals, which can limit detection performance.^134^ The adoption of simple, one-step carbonization process using renewable tea waste is expected to yield low-cost, carbon-rich waste materials for fluorescent CD synthesis.^135^ CDs facilitate the detection of free chlorine in water systems *via* fluorescence burst methods, offering high sensitivity, selectivity, rapid detection, and cost-effectiveness within a green framework.^136^ For instance, CDs produced at 700 °C from waste from bitter tea oil extraction achieved a maximum quantum yield obtained of 11.8%. CDs synthesized for Fe^3+^ detection exhibited a LOD of 0.380 ppm, attributed to their graphitic structure, aromatic stability, and efficient π–r* electron transitions.^137^ WTPs have also been used as C and N sources to generate borazine-carbon dots (BN-CDs), which exhibit blue luminescence due to boric acid as the boron source. Boron doping in BN-CDs introduces p-type charge carriers, modifying the electronic structure and fluorescence quantum efficiency by altering the internal filtering effect. Specifically, electrons from excited BN-CD states transfer into the 3d-orbitals of Fe^3+^, resulting in fluorescence quenching *via* an internal filter effect. This mechanism enables BN-CDs to act as selective probes for Fe^3+^ detection, with a linear relationship observed between fluorescence quenching efficiency and Fe^3+^ concentration.^138^ In a related study, Al-Hazmi *et al.* (2022), synthesized Fe_3_O_4_*via* co-precipitation method from FeCl_3_ and urea, subsequently combining it with AC derived from biomass through pre-carbonization and potassium hydroxide activation.^139^ The resulting Fe_3_O_4_/AC composite exhibit varying adsorptive capacities for degrading four different dyes (Dye 1, 2, 3, and 4) under diverse pH conditions, composite dosages, and contact times, with capacities of 238.6 mg g^−1^, 192.7 mg g^−1^, 304.0 mg g^−1^, and 286.5 mg g^−1^, respectively. These findings indicate that the Fe_3_O_4_-carbon composite is an effective adsorbent for detoxifying dyed water sources. In addition, the composite demonstrated eco-friendliness, cost-effectiveness, reusability, and significant potential for the removing organic dyes.

Reported adsorption capacities vary greatly among studies due to differences in experimental conditions—initial concentration of contaminant, solution pH, temperature, contact time, and dose of adsorbent. Direct comparison of the adsorption capacities of WTP-derived materials and commercial adsorbents is not advisable without normalization of these two materials. Furthermore, most studies reporting high adsorption capacities do not account for realistic operational conditions, resulting in inconsistencies when comparing adsorbent performance. Consequently, the direct translation of laboratory data to real-world applications remains limited. To enable accurate comparisons between WTP-derived compounds and commercial counterparts, a standardized assessment protocol for adsorption should be established.

### 5.3 The adsorption bottleneck

Previous sections have outlined the notable characteristics of WTP-CNMs. However, it is important to emphasize that adsorption is a physical process governed by thermodynamic equilibrium. Consequently, both the physical limitations of active sites and the environmental impact of waste NMs fundamentally define the Adsorption Bottleneck.

WTP-derived adsorbents are constrained by the saturation of their surface functional groups. For BR46, MB, and MO, adherence to the Langmuir isotherm model indicates that, after the initial monolayer of dye molecules forms on the adsorbent, insufficient thermodynamic driving force remains to facilitate further adsorption.^53,119,140^ The Intraparticle Diffusion Model (IDM) quantifies this process. Jóźwiak *et al.* (2021) demonstrated three distinct phases in the adsorption of cationic dyes: (1) rapid uptake due to surface-film diffusion, (2) slower uptake governed by intraparticle diffusion, and (3) a plateau phase where concentration remains unchanged.^140^ This plateau is commonly referred to as the “Saturation Bottleneck”. In an industrial environment, once WTP-CNMs exceed their maximum adsorption capacity (*Q*_max_), such as 179.4 mg g^−1^ for BR46, their subsequent dye removal capacity becomes negligible.^140^ To sustain continuous operation, large quantities of novel WTP-CNM must be introduced. In laboratory studies, the use of WTP-CNM has enabled assessment of material performance under conditions relevant to commercial applications, even when limited adsorbent has precluded full assessment of their primary advantages. The most significant “Beyond Adsorption” consideration is the environmental impact of disposing of saturated WTP-CNM. Traditional adsorption is a phase-transfer mechanism; it does not degrade the pollutant but instead transfers it from the liquid phase (wastewater) to the solid phase (spent adsorbent). As a result, this process lead to secondary pollution.

Dye-contaminated WTP-CNMs and pharmaceutical contaminants, including hydralazine hydrochloride, are classified as hazardous waste.^141,142^ Improper disposal of these materials could pose additional environmental risks, including partial desorption, leaching, and potential release of contaminants.^1,2^ Non-covalent bonds, including electrostatic and π–π stacking interactions, that retain contaminants can degrade under environmental changes, potentially allowing toxic substances to re-enter groundwater.^111,143^ The linear adsorption model, based on quantitative analysis, is insufficient for large-scale decontamination of textiles or pharmaceuticals.^144^ The transition to the catalytic mineralization process represents both an important advancement in science and an urgent necessity.^145^ WTP-CNMs can be transformed into renewable catalytic systems that will help address “Adsorption Bottlenecks” and disposal difficulties facing traditional adsorbents, thus encouraging the development of circular economies for waste-to-treatment systems.

Adsorption is limited by phase transfer and the lack of transformation of contaminants, thereby requiring additional processes to degrade pollutants.^146^ Overall, although adsorption of pollutants onto WTP-CNMs is generally effective, limitations exist due to saturation and phase transfer; therefore, it is necessary to adopt catalytic techniques to mitigate these issues.

## 6. Catalytic degradation mechanisms and material design

Catalytic degradation mechanisms are classified into three distinct categories based upon the method of activation: (i) radical-based (PDS and PMS), providing activation through the generation of SO_4_˙^−^ and ˙OH radicals; (b) non-radical pathways that generate singlet oxygen and induce oxidation; and (c) photosensitized systems/photochemical energy transfer systems involving chromophores. Additionally, unlike adsorption, where the pollutants are merely transferred to another phase, catalytic degradation transforms pollutants into less toxic or mineralized products through either radical or non-radical pathways using reactive species. As such, catalytic degradation alleviates the major limitation of adsorption by facilitating pollutants destruction compared to simple phase transfer. Recent studies have reported the development of multifunctional catalytic adsorbents, which include defect-engineered carbon materials and heteroatom-doped nanostructures that provide the ability to adsorb and degrade simultaneously. The function of these systems revolves around a synergistic mechanism where the pollutants first reside at the surface of the adsorbent and are then degraded either through radical or non-radical pathways, thereby significantly improving both the efficiency of removal and reusability of the material.

Adsorption refers to the physical removal of contaminants from the environment *via* sorption onto a surface, whereas catalytic degradation involves the chemical decomposition of contaminants into simpler forms *via* chemical reactions.^147,148^ In catalytic degradation, WTP-CNMs function as reactors rather than passive sinks.^149^ The transition from sorption to chemical sequestration is closely associated with a contaminant's ability to transfer electrons and generate ROS molecules during chemical degradation.^150,151^

WTPs, a nitrogen-rich lignocellulosic agricultural waste, serves as a sustainable and cost-effective precursor for synthesizing metal-free CNMs. Table 3 summarizes various catalytic systems employing WTP-derived CNMs, detailing the oxidants used, associated mechanisms, and corresponding degradation efficiencies. In AOPs, CNMs activate PMS and PDS for the effective removal of dyes.^161^ N-doping within these carbon structures is critical, as it introduces electron-rich sites, such as pyridinic-N and graphitic-N, which influence the charge density and spin distribution of adjacent carbon atoms.^162,163^ This modification, depending on catalyst structure, oxidant type, and reaction conditions, promotes both radical pathways (including sulfate, hydroxyl, and superoxide radicals) and non-radical pathways (such as singlet oxygen generation and electron transfer) pathways.^163,164^ Recent studies have demonstrated the efficiency of N-doped WTP-CNMs in activating persulfates for degrading several organic contaminants, including dyes.^165^ As an illustration, WPRN700 synthesized from the pyrolysis of heat paste residues and urea, functions as a cost-effective and environmentally friendly catalyst. This material achieved rapid catalytic degradation of bisphenol A (BPA) *via* PMS activation, with BPA removal rates reaching up to 99% within 30 min at an initial BPA concentration of 20 mg L^−1^.^166^ Similarly, N-doped porous carbon nano-filters, fabricated from cellulose nano-fibres and metal–organic frameworks (MOFs), have been shown to be effective metal-free catalysts for sulfate radical-based AOPs.^167^ Studies have confirmed that N-doped porous CNMs and the activation of PMS/PDS are both effective in removing a range of organic contaminants, including azo dyes such as AO7, MO, and MB.^163,168^ Previous research reported dye removal efficiency of 90% within 10–30 min.^161,169^ Additional investigations have demonstrated that N-doped carbon nanosheets derived from waste materials effectively activate PMS for the degradation of AO7.^169^ Furthermore, N-doped porous aluminium produced by pyrolyzing polyacrylonitrile has shown high efficiency in activating PMS for the degradation of organic contaminants in water.^170^

**Table 3:** Catalytic dye degradation systems based on WTP-derived CNMs: oxidants, mechanisms, and degradation efficiency

Catalyst (WTP/tea-derived)	Activation system/support	Oxidant	Target (dye/pollutant)	Removal (%)	Time (min)	Dominant ROS/species reported	Mechanism type	Reusability (cycles)	References
Fe_3_O_4_-loaded tea-residue biochar (Fe_3_O_4_@T-BC)	Heterogeneous magnetic catalyst	PMS (peroxymonosulfate)	Tetracycline hydrochloride (model organic; transferable to dyes)	97.9%	60	SO_4_˙^−^, ˙OH (EPR/quenching evidence)	Radical PMS activation (heterogeneous)	Recycled 4× (71% after 4th run)	152
Spent-tea-leaves biochar (metal-free STLB)	Adsorption-enhanced PS-AOP (biochar as PS activator)	Persulfate (PDS/PS)	Chlortetracycline (CTC)—model pollutant (adsorption + degradation)	≈97.4% (pre-adsorption 30min + 60 min degradation)	90 (30 + 60)	SO_4_˙^−^, ˙OH and non-radical ^1^O_2_ (dominant non-radical observed)	Adsorption-promoted persulfate activation (radical + non-radical)	Good recyclability reported (wide pH 3–9)	153
CeO_*x*_-modified tea biochar (Ce-TBC)	Metal-oxide modified biochar for PDS activation	PDS (peroxydisulfate)	Tetracycline (TC)	91.3% (under optimal conditions)		^1^O_2_ (non-radical dominant), plus radicals detected	PDS activation *via* Ce^3+^/Ce^4+^ redox + ^1^O_2_ pathway	∼5 cycles (≈80% after 5)	154
Spent tea-leaf biochar (STLB) used as adsorption-enhanced PDS activator (metal-free)	Adsorption follow by PS activation (biochar-promoted)	PDS/PS	Tetracyclines/refractory organics	>95% (reported for tetracyclines in several tea-biochar studies)	Variable (30–60)	SO_4_˙^−^, ^1^O_2_, ˙OH (mixed radical & non-radical pathways)	Adsorption-enhanced PS activation (metal-free)	Reusability described (multiple cycles)	153
Tea-leaf-extract synthesized nano-ZVI (nZVI)	Fenton/electro-Fenton style (Fe^0^ → Fe^2+^/Fe^3+^)	H_2_O_2_ (Fenton reagent)	Mixed dyes (rhodamine B + methyl orange)	RhB 100%; MO ∼66.5% (LC-MS)	60	˙OH (Fenton hydroxyl radicals)	Green nZVI + Fenton oxidation	Noted performance; reusability specifics	155
Magnetic porous carbonaceous material from tea waste (γ-Fe_2_O_3_ anchored MPC)	Magnetized porous carbon with *in situ* iron oxides	No external oxidant (adsorption)/or used as support for oxidant activation in related work	Methyl orange (MO), other dyes (as tested)	High removal of MO (reported qualitatively high adsorption; adsorption-favorable)	Short (adsorption kinetics fast)	Adsorption dominant (but iron sites enable further catalysis in modified forms)	Adsorption; can be adapted as Fenton/PS activator if loaded/modified	Magnetic separation eases reuse; cycles reported in paper	156
NaOH-modified mesoporous tea biochar (BH700-10 type)	Chemical activation (NaOH) to create mesopores and graphitic domains	(Used as adsorbent; also favorable electronic structure for PS activation	Methylene blue (MB) and orange II (OR-II)	High removal for MB & OR-II (reported as efficient; adsorption dominated)	60 (typical adsorption tests)	Adsorption + possibility for electron transfer to oxidant	Adsorption; potential for adsorption-promoted AOP	Regeneration tests reported	157
Tea-derived activated carbon supporting Ag–TiO_2_ nanoparticles (g-AC@Ag–TiO_2_)	Carbon support improves charge separation of TiO_2_ photocatalyst	Light (UV/solar) (photocatalysis)	Methylene blue (MB)/rhodamine B (typical dyes for these composites)	>90% (photodegradation reported for TiO_2_@C composites in related work)	Tens of minutes to hours under UV/solar	˙OH, O_2_˙^−^ (photogenerated radicals)	Heterojunction photocatalysis (carbon support enhances separation)	Photocatalyst reuse	158
Biochar-supported zero-valent copper/copper-based species on tea biochar	Biochar support + zero-valent/oxide metal for PMS/PDS activation	PMS/PDS	Model organics (antibiotics, dyes)	High degradation (near complete for some targets in <60 min)	∼30–60	SO_4_˙^−^, ˙OH and electron-transfer routes	Heterogeneous metal-mediated persulfate activation	Reusability depends on metal leaching; many report multiple cycles with moderate loss	159
Mn/Ce/MnCeO_*x*_-modified tea biochar (metal-modified)	Metal-oxide modified biochar for persulfate activation	PDS/PS	Tetracycline/other organics	80–95% under optimized conditions (paper-dependent)	30–90	Non-radical ^1^O_2_ often reported with Ce; radicals for Mn	PDS activation *via* redox cycles (Mn/Ce) & oxygen vacancies	Reuse reported (some decline over cycles)	154
Tea-derived carbon quantum dots (CQDs) combined with TiO_2_	Carbon quantum dots (from tea) + TiO_2_ photocatalyst	Light (visible/solar)	Methylene blue (MB)/model dyes	Strongly enhanced photodegradation *vs.* TiO_2_ alone (many reports >90%)	30–120 under visible/solar	˙OH, O_2_˙^−^ (photogenerated)	Photocatalysis with improved visible response	Photocatalyst stability tested (multiple runs)	160
Tea-waste activated carbon (KOH or chemical activated) impregnated with Fe (Fe/AC)	Chemical activation + Fe impregnation for Fenton-like or PS activation	H_2_O_2_ or PS	Dyes (MB, RhB) and organics	High removal; many studies report >85–95% under optimized conditions	30–120	˙OH (Fenton) or SO_4_˙^−^ (PS)	Heterogeneous Fenton/PS activation	Reusability: variable; magnetic or immobilization helps	42
Tea residue biochar/tea waste composites combined with transition metal oxides (*e.g.*, Fe_3_O_4_, MnO_2_)	One-pot impregnation/pyrolysis → magnetic/metalized biochar	PMS/PDS/H_2_O_2_ depending on study	Dyes (MO, MB, RhB) and model organics	Many reports show >80–98% under optimized lab conditions	20–120	˙OH, SO_4_˙^−^ and sometimes ^1^O_2_ (mixed)	Heterogeneous metal-mediated activation or photocatalysis (if combined with light)	Reusability tested in multiple papers (magnetic recycling common)	156 and 157

The performance of WTP-CNMs in the real world has not been adequately characterized to date, while most recent reviews frame adsorption and catalytic degradation as two distinct processes. This review combines both mechanisms within a single framework and also discusses post-treatment soil applications, providing a much better understanding of how tea-residue-derived CNMs can be utilized. In addition, there is little data quantifying how various factors affect the catalytic performance of WTP-derived CNMs, such as heteroatom composition, defect density, and surface electronic structure, which influence the formation of reactive species and electron transfer mechanisms.

Catalyst stability and reusability are essential for practical catalytic applications. N-doped WTP-CNMs demonstrate high stability across multiple cycles (>5 runs) exhibit minimal N leaching (<0.1 mg L^−1^), and possess robust physical structures, making them promising alternatives to transition-metal-based catalysts.^161^ However, certain limitations persist, such as catalyst deactivation resulting from radical grafting onto the carbon surface, particularly during BPA treatment with PMS activation.^171^ In addition, the presence of structural defects and the specific type of N-doping, such as graphitic N and pyridinic N, significantly influence catalytic performance and stability. While these materials are cost-effective and environmentally friendly, the activation mechanisms of metal-free catalysts, such as N-doped CNMs, differ fundamentally from those of transition metal-based catalysts.^172^ For instance, metal-containing catalysts (like Fe, Co, or Mg) typically utilize metal centers as primary active sites for redox cycling and electron transfer with persulfates.^173,174^ In contrast, N-doping in carbon matrices increases the number of available active sites, enhances electrical conductivity, and regulates ROS formation.^175^

Understanding the roles of various N-species in the activation process has advanced significantly. Some studies suggest that although ^1^O_2_, a common N-species in AOPs involving persulfate, may not play a major role in the removal of organic components, the quenching methods used to detect it may not always accurately reflect its presence.^176,177^ Recently, active sites on carbon surfaces and specific N-configurations have attracted increased attention due to their essential roles in electron transport processes.^178,179^ Therefore, the rational design of N-doped CNMs derived from WTPs represents a sustainable, cost-effective approach to enhance wastewater remediation.

While adsorption-based approaches provide initial pollutant removal, their limitations necessitate a transition toward catalytic degradation strategies, as discussed in the following section. Beyond aqueous remediation, the multifunctional properties of WTP-derived CNMs enable their application in soil systems, where both adsorption and catalytic processes contribute to pollutant mitigation.

### 6.1 Photosensitized and light-driven catalytic degradation pathways

In addition to persulfate-based activation mechanisms, a growing body of literature has focused on the role of photosensitized catalytic systems for pollutant degradation. Organic chromophores such as xanthene-based dyes can act as the photosensitizer under visible light irradiation, generating reactive species, including singlet oxygen (^1^O_2_), superoxide radical (O_2_˙^−^), and excited triplet state *via* energy transfer and electron transfer mechanisms. Photosensitized catalytic systems therefore extend beyond traditional radical-driven systems. Current research has shown that the tunable photo-sensitizing characteristics exhibited by xanthene chromophores are affected by halogen substitution and molecular structure—two factors capable of affecting both the triplet-state generation and the ROS production. Thus, it appears that sunlight-driven degradation of contaminants can be achieved through the use of non-radical and selective oxidative processes as opposed to persulfate activation mechanisms.^180^ While advantages such as less chemical input, solar energy utilization, and more selective processes might exist for photo-sensitized processes when compared with persulfate activity. However, their efficiency remains limited by factors such as the intensity of the light source, chromophore stability, and matrix effects—indicating that further optimization is required.

### 6.2 Pathway 3: water treatment and soil detoxification

The sustainable recycling of WTP-CNMs offers a long-term, multi-functional approach to environmental remediation following aqueous treatment.^181^ The residual properties of WTP-CNMs can further address two major issues in soils converted into biochar-based nanocomposites: heavy metal contamination and organic pollutant degradation.^182^ Biochar, the carbonized product of biomass pyrolysis, is inorganic and highly porous, with a large surface area and significant cation-exchange capacity. These properties enhance soil quality and improve contaminant retention.^14,183–185^

The immobilization of heavy metals in contaminated soil using WTP-CNMs and biochar-based nanocomposites has been shown to effectively reduce their leachability and uptake rates.^181,186^ This process primarily occurs through chelation, surface complexation, and precipitation. WTP-CNMs possess a high degree of surface functionality due to –COOH and –OH functional groups, as well as N-doped carbon sites that facilitate solid-to-solid interactions with various heavy metal ions, including Pb^2+^, Cd^2+^, Cu^2+^, and As(iii/v).^187^ The presence of tea polyphenols, such as catechins and tannins, in WTPs further enhances the chelating capacity of WTP-CNMs for these metal ions.^188^ The binding of these metal ions limits their migration within the soil profile, accumulation in plants, phytotoxic effects, and entry into the food chain.^182,189^ Evidence indicates that the immobilization effect of heavy metals (*e.g.*, Cd, Cu, and Pb) in contaminated soils can be significantly enhanced by applying biochar, with effects persisting for years after application.^190,191^ In unsaturated soils, the adsorption capacities of biochar for newly contaminated heavy metals follow the order Cd > Ni > Cu.^192^ Removal of heavy metals, including Cd, from contaminated soils is highly effective when using metal oxide-engineered biochar, such as rice husk biomass encapsulated with manganese dioxide (MnO_2_), which demonstrate superior Cd removal compared to other materials.^193^ The interaction between cellulose with hemicellulose on the surfaces of biochar or biomass and metal oxides is critical in determining the stability of metal loading and the potential for adsorption. The porous structures of WTP-CNMs and biochar support microbiological activity and facilitate the immobilization of heavy metals, thereby enhancing the efficiency of bioremediation treatments. Stimulation and augmentation of both bio-stimulation and bioaugmentation processes can increase the degradation rates of residual organic contaminants in soil. Table 4 summarizes current research findings on the application of these nanostructured materials for post-treatment reuse, highlighting their dual roles in soil remediation and microbial growth stimulation.

**Table 4:** Post-treatment reuse of WTP-derived CNMs for soil remediation and bio-stimulation applications

CNM source (tea-derived)	Contaminant type	Soil/matrix type	Remediation mechanism (authors' description)	Improvement observed (key numbers)	Biological impact reported	Study scale	References
Tea waste biochar (TWBC)	Cadmium (Cd)	Contaminated sediment (meso-microcosm)	Adsorption/immobilization to biochar surfaces; shift of Cd from exchangeable → less available fractions	Exchangeable Cd fraction reduced ≈67.7%; reduced uptake in plants/molluscs (root/shoot reductions 75–87% in Eichhornia)	Lower bioaccumulation in biota; improved sediment quality indicators	Lab meso-microcosm (sediment)	194
Tea pruning litter biochar (TPLBC)	As, Cd, Cr (multiple trace metals)	Field tea-garden soil (*Camellia sinensis* plantation)	Immobilization/fractionation shift (increase in residual fraction); pH and CEC modification reduces mobility	Reduced As, Cd, Cr in made tea (leaf) and infusion; ADI & hazard quotients significantly lowered (*p* ≤ 0.01)	Improved food safety (lower metal in harvested product); soil micronutrient balance altered (Cu, Mn, Zn)	Field trial (0–360 days)	195
Tea leaves biochar co-applied as carrier for Bacillus cereus (biochar + PGPR)	General soil fertility/not target heavy metal only	Agricultural topsoil (mung bean field/pot experiments described)	Biochar acts as microbial carrier & microhabitat; enhanced colonization, nutrient retention and enzyme activity	Soil organic C, microbial biomass C & N, Olsen-P increases (percent increases reported, *e.g.*, SOM, AN, AP improved)	Enzyme activities ↑ (urease, dehydrogenase, phosphatase); increased crop yield and N_2_ fixation	Field/pot experiments (applied in agricultural plots; multi-season)	196
Green tea biochar supported nZVI (nZVI@GTBC)	Lead (Pb) (and general metal mobility)	Contaminated agricultural soil (lab incubation/mechanistic study)	nZVI reduction + biochar adsorption/complexation; pH and redox changes → precipitation and sequestration	nZVI@GTBC improved Pb immobilization by ∼19–57% *vs.* pristine controls; increased residual (stable) fractions	Lower Pb bioavailability; improved soil pH & SOM; longer-term immobilization compared to nZVI alone	Lab incubation & mechanistic study (environment int. 2020)	197
ZVI/ZVI-decorated tea biochar (ZVI@TBC) (zero-valent iron on tea biochar)	Cd(ii) and Cr(vi)	Aqueous/sediment and soil application tests (lab)	Reductive transformation (Cr vi → Cr iii), adsorption and co-precipitation on iron oxidation products anchored to biochar	High Cd/Cr removal in water tests; in soil/sediment, reduced mobility and speciation change to less available forms (authors report high % removal in batch tests)	Reduced metal mobility and bioavailability; authors discuss leaching & reusability concerns	Lab studies; batch and soil tests reported	198
Tea-derived biochar supported nZVI (BC-nZVI) (porous biochar + nZVI)	Cd, Pb	Clayey/contaminated soils (lab soil remediation tests)	Adsorption + reduction + precipitation (BC supports nZVI, reduces aggregation and offers adsorption sites)	BC-nZVI immobilized Cd & Pb ∼80–90% in some tests; increased soil pH & SOM	Reduced extractable fractions; improved short-term soil properties	Lab soil remediation experiments (W. Qian *et al.*, 2022 style studies)	199
Acid-modified tea-waste biochar	Cr(vi) or nutrient/metal interactions (in crop test)	Pot experiments/greenhouse soil (*Allium cepa* growth tests)	Surface chemistry modification to increase active sites for adsorption/immobilization; pH/CEC effects	Improved plant biomass and reduced metal uptake in some treatments; altered uptake dynamics	Changes in root/shoot growth metrics; potential amelioration of phytotoxic effects	Pot experiments/greenhouse	200
Metal-modified tea biochar (Mg/Fe/Mn/Al salts)	Phosphates/heavy metals interaction (nutrient immobilization context)	Soil/water matrices (lab tests)	Metal salt modification enhances cation exchange and adsorption sites; improves phosphate removal and metal immobilization	Enhanced adsorption capacity for PO_4_ and improved immobilization metrics *vs.* unmodified biochar	Authors report altered soil nutrient availability; potential to alleviate metal toxicity indirectly	Lab experiments & batch tests	112
Tea pruning litter biochar (TPLBC)	Micronutrient (Cu, Mn, Zn) dynamics & contamination risk	Tea plantation soils (field/multi-dose trials)	Alters metal fractionation, improves CEC and pH, and changes micronutrient availability (stabilization)	TPLBC influenced availability and distribution of Cu, Mn, Zn; recommended dosing (*e.g.*, 400 kg ha^−1^) for benefits	No adverse ecological risk according to geo-accumulation, enrichment, and risk indices in the study	Field assessment (0–360 days)	201
Tea leaves/tea waste biochar + plant growth tests	Glyphosate uptake/organic contaminant transport	Pot/field tests (maize irrigated with glyphosate-contaminated water)	Biochar adsorption of glyphosate; improved nutrient retention & reduced glyphosate bioavailability	Improved maize growth and reduced glyphosate uptake compared to control (specific % reductions in tissue reported in study)	Increased crop resilience, improved nutrient uptake	Pot/field scale (greenhouse/field trial)	202
Tea waste-derived carbon used as a microbial carrier/habitat	Not a single contaminant—applied to contaminated soils to enhance biodegradation	Agricultural/contaminated soils	Provides porous habitat for microbes, supports bioaugmentation/bio-stimulation, increases enzyme activity	Increased microbial biomass, enzyme activities (urease, dehydrogenase), improved nutrient cycling in multiple studies	Strong increases in microbial abundance and activity; improved plant growth where tested	Multiple lab & field studies surveyed (review evidence)	203
Tea-derived carbon used to prepare green-synthesized iron nanoparticles/nZVI	Dyes/metals (lab remediation tests)	Soil/water batch experiments & soil amendments (lab scale)	Tea polyphenol-mediated synthesis of nZVI; nanoparticles anchored/combined with biochar for adsorption + reduction	High removal of model contaminants (dyes/Cr/Cd) in lab tests; efficiency depends on dosage & aging	Authors report reduced contaminant bioavailability; discuss nanoparticle fate & eco-toxicity	Lab batch and small soil incubation studies	204
Spent-adsorbent derived biochar (general: post-adsorbent → biochar)	Cu, Cd (post-adsorbent used for *in situ* immobilization)	Contaminated lake sediment/soil microcosms	Re-valorization: adsorbent loaded with metal then pyrolysed → reused as *in situ* immobilizer (adsorption + co-precipitation)	Reduced extractable metal fraction and *in situ* immobilization in sediment/soil; demonstrated as circular reuse route	Lower bio uptake in test organisms; authors discuss safety & leaching concerns	Sediment microcosm/lab tests (pilot scale)	205

Both indigenous and inoculated microorganisms, such as *Pseudomonas* spp. and *Bacillus* spp., can thrive within the high surface area and protected environments created by the meso- and macro-porosity of WTP-CNM biochar. The porosity and the structure of WTP-CNMs facilitate direct electron exchange between isolated microorganisms, thereby enhancing the rate and effectiveness of degradation by anaerobic bacteria. These microhabitats provide ample surface area for initial microbial attachment and subsequent growth.^206^ Increased microbial activity has been shown to promote the degradation various organic pollutants, including polycyclic aromatic hydrocarbons (PAHs) and chlorpyrifos.^207,208^ The gradual release of carbon and nutrient compounds from WTP-CNMs promotes biostimulation by enhancing the activity of resident microbial populations. For instance, biostimulation with ammonium nitrogen accelerates degradation of phenanthrene in oil-contaminated soils. Nutrient additions, such as N and P, along with the provision of electron acceptor, such as oxygen, can further stimulate existing microbial communities to degrade contaminants more rapidly.^209,210^ The physicochemical properties of WTP-CNMs also supports bioaugmentation, in which specific microbial populations are introduced into contaminated environments.^211^ The highly porous nature of WTP-CNMs creates optimal conditions for these inoculated microorganisms to establish and proliferate, leading to increased colonization, increased extracellular polymeric substance (EPS) production, and improved electron transfer efficiency. Bioaugmentation is particularly effective when indigenous microbial communities lack the metabolic pathways required to degrade specific contaminants or when native degraders are too slow to establish a dominant population. For instance, biostimulation of a fungal consortium with nutrient additions has resulted in significant reductions of total petroleum hydrocarbons (TPH) in diesel oil-contaminated soil.^212^

**Fig. 3:**
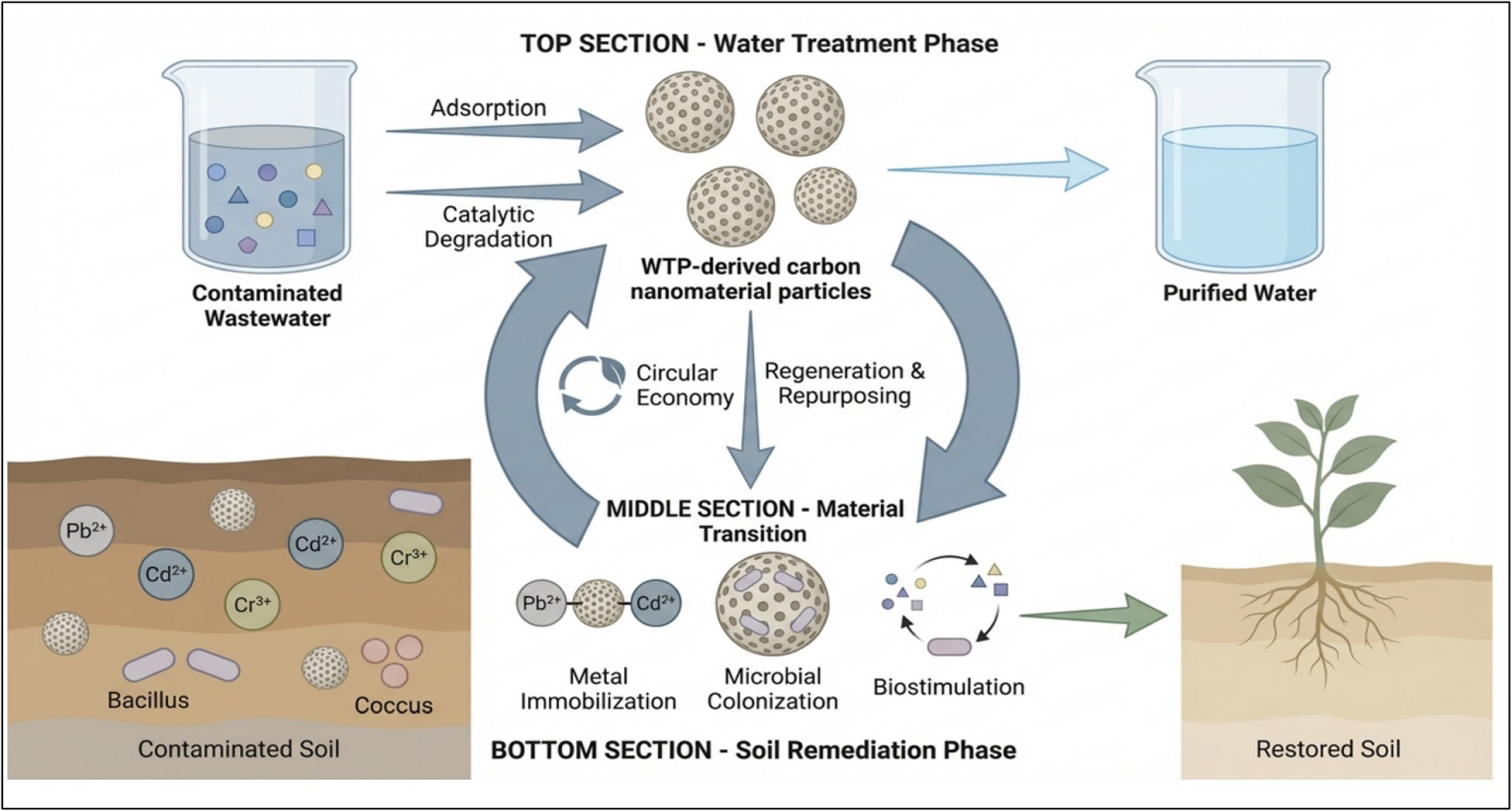
Integrated water–soil remediation pathway using multifunctional WTP-derived CNMs. Following dye adsorption and catalytic degradation in wastewater treatment, regenerated or spent WTP-derived CNMs can be repurposed for soil remediation. In contaminated soils, these materials immobilize heavy metals through surface complexation and chelation, while their porous structure supports microbial colonization and bio-stimulation. This integrated water–soil remediation framework highlights the circular reuse potential of WTP-derived CNMs and their role in sustainable environmental management.

WTP-CNMs have been shown to enhance the removal of residual organic contaminants from soil by increasing the activity of enzymes responsible for laccase and peroxidase production. When applied at low nutrient or redox mediator concentrations, WTP-CNMs promote syntrophic interactions among microbial populations. This process is illustrated in Fig. 3, which demonstrate a ‘Capture and Kill’ synergistic mechanism: dye molecules are sequestered within the hierarchical pores of the WTP-CNMs, while N-doped defects generate radicals that facilitate mineralization. Field and microcosm studies have demonstrated that the combined application of WTP-CNMs significantly reduces metal phytotoxicity and decreases the half-lives of organic pollutants.^213,214^ Collectively, WTP-CNMs function as a dual-action, cyclical, multi-functional additive that enables efficient soil remediation. The integrated mechanism of WTP-CNMs provides a novel, effective long-term solution for the safe remediation of extremely contaminated sites. Recent studies have shown that the combined use of adsorption and catalytic degradation provides a synergistic method whereby pollutants are first concentrated on the adsorbent material surface and then subjected to a subsequent catalytic phase.^184,215,216^ This “adsorption–degradation coupling” overcomes mass transfer limitations and improves overall efficiency compared with using these two processes separately.

Some major research gaps still exist despite recent advancements. Mechanistic understanding of adsorption–degradation coupling with regard to how surface chemistry interacts with electron transfer and the reactive species generation is not well understood. Current research has mostly been conducted in a controlled laboratory setting, with limited validation in real wastewater or soil systems. Additionally, there are challenges with material stability, regeneration and scalability that can hinder application. To further develop the next generation multifunctional remediation systems, the above research gaps must be addressed.

## 7. Critical gaps

WTP-CNMs show great potential as environmental remediation technologies. However, there are several limitations in our current knowledge and challenges to their practical use. This section proposes a long-term research agenda, informed by a review of existing literature and a defined research path, with particular emphasis on the characteristics of most impactful studies. The current WTP-CNMs literature lacks standardized methods for preparing WTP-CNMs.^181^ Variability in the source type of WTP (*i.e.*, green tea, black tea, or oolong tea) and in preparation methods (including drying techniques) results in differences in the chemical and physical properties of CNMs produced from WTP.^4,5^ Additionally, variations in polyphenols, lignin, and cellulose content among tea components affect the yield of carbonized CNMs, the size and shape of pores, surface functionalization, and ultimately the effectiveness of CNMs in degrading pollutants. The development of WTP-CNMs with broad applicability and reproducibility is hindered by the inability to compare data across different WTPs sources and types, primarily due to the lack of standardization. Researchers are currently assessing variations between raw WTPs and synthesized CNMs using holistic approaches, including elemental analyses, surface area measurements, and spectroscopic techniques. However, these efforts do not address the fundamental issue of standardizing raw WTPs used for CNM production.^181^ Future research should prioritize addressing WTP-CNMs techniques that either lack or mitigate the effects of WTPs variability, such as through pre-treatment standardization or WTP-independent synthesis methods. Another major barrier to implementing advanced CNM synthesis technologies is their cost-effectiveness, particularly when using methods such as chemical vapour deposition (CVD) or enhanced hydrothermal processing techniques.^217,218^ While these techniques enable the design of CNMs with highly specific structures and optimal laboratory performance, they are energy-intensive, require costly equipment, and are constrained by small-batch processing and high long-term costs. Consequently, current research often fails to assess the cost-effectiveness of innovative synthesis techniques in the context of industrial scale-up. As the field moves toward industrial scale applications, it is essential to develop low-cost, high-throughput processes that utilize readily-available, less expensive raw materials, aligning with the waste valorisation objectives of WTP-CNMs.

## 8. Challenges/limitations (life cycle assessment and sustainability considerations)

A major challenge in assessing the sustainability of WTP-derived CNMs is the lack of comprehensive Life Cycle Assessment (LCA) studies evaluating the environmental effects of CNM production and use in all life cycle stages, including synthesis, application, regeneration, and disposal. Most existing studies focus primarily on laboratory-scale material properties and provides limited date on energy consumption, chemical inputs, and potential secondary environmental impacts associated with large-scale production or application of these materials.

Recent trends in LCA research point toward a growing collaboration between different research communities and disciplines across the science and engineering sectors.^219^ Increased focus will be placed on assessing the carbon footprint of products, examining resource efficiency, and conducting system-level sustainability evaluations. Key priorities include improving data quality and availability, developing methods to evaluate uncertainty, and integrating environmental, economic, and circular economy indicators to create holistic assessment approaches.^219^ The advances underscore the need to move from simplified performance-based assessments towards more comprehensive sustainability metrics. Assessing the environmental benefits of the CNMs by considering the effects of precursor processing, activation, and post-use disposal or regeneration will be a critical step towards enabling scalable and sustainable implementation of tea-residue-derived NMs.

## 9. Future directions

Based on limitations and challenges in previous sections, several major research areas have emerged to advance the development of tea-residue-based CNMs for environmental remediation. A primary focus should be the establishment of standardised methods for determining the adsorption capacities of CNMs derived from WTPs, considering experimental parameters such as pH, initial pollutant concentration, adsorbent dosage, and contact time. Standardising the evaluation of WTP-derived CNMs will enable more reliable performance assessments and facilitate direct comparisons with commercial adsorbents. Further research is also needed to address the limited understanding of the chemistry underlying catalytic degradation of pollutants, particularly in adsorption–degradation coupling systems. Elucidating the role of surface functional groups, defect sites, and doped heteroatoms in electron transfer and reactive species generation is a key priority. Employing advanced kinetic modeling and *in situ* spectroscopic techniques may provide new insights into reaction pathways. As most studies have been conducted under controlled laboratory conditions, subsequent research should evaluate the effectiveness of WTP-derived CNMs in real environmental matrices, such as wastewater treatment systems and soil. Material performance and stability are influenced by factors including competing ions, natural organic matter, and altered environmental conditions. Integrating photo-responsive functions into WTP-derived CNMs offers a promising strategy for developing catalytic mechanisms that go beyond conventional processes and persulfate activation methods. Furthermore, photochemical activation of WTP-derived CNMs advances sustainable practices by utilizing solar-based energy sources rather than chemical sources. Ongoing challenges associated with material scalability, regeneration, and long-term environmental impact require continued investiagtion. Future studies should address the life-cycle performance and safe, sustainable deployment of WTP-derived CNMs. These research priorities are essential for translating laboratory advances into practical, scalable, and environmentally sustainable remediation technologies.

## 10. Conclusion

Materials derived from WTPs represent a significant advancement over traditional adsorbent-based materials, as they offer both adsorption (capture) and catalytic (degradation) functionalities for contaminant removal. The functional properties of WTP-derived CNMs can be improved through heteroatom doping, defect engineering, and hierarchical porosity. These modifications facilitate electron transfer, thereby enabling both radical and non-radical degradation pathways. The integration of adsorption and catalytic processes offers potential solutions to several of the major limitations of adsorption, such as saturation and phase-transfer constraints, by improving contaminant removal efficiency and sustainability. This multifunctionality supports the application of WTP-derived CNMs in both aqueous and soil remediation, aligning with a circular waste-to-resource approach. Despite these advancements, challenges persist regarding mechanistic understanding, material stability, scalability, and field-level validation. Future research should focus on the development of standardized synthesis protocols, clarification of of adsorption–degradation coupling mechanisms, and validation of these materials in real-world applications. While current findings demonstrate considerable promise, further validation through real-world and standardized testing is necessary to substantiate performance claims. The development of multifunctional CNMs from waste biomass thus presents a promising pathway toward sustainable and scalable environmental remediation.

## Author contributions

Shareen Niyazi: writing – original draft; writing – review & editing; conceptualization; software, resources, supervision. Mohammad Shahid: data curation; resources; writing – original draft. Aman Raj: formal analysis; software; writing – original draft.

## Conflicts of interest

The authors report no potential conflicts of interest including financial or personal relationships that could have influenced this research.

## Data Availability

No primary research results, and no new data were generated or analysed as part of this review.
